# Genetic variants in *HELB* contribute to premature ovarian insufficiency and early age of natural menopause

**DOI:** 10.1172/jci.insight.191122

**Published:** 2025-11-10

**Authors:** Yuncheng Pan, Yuexin Yu, Jitong Mo, Shuting Ren, Zixue Zhou, Xi Yang, Yiqing Liu, Feng Zhang, Yanqin You, Xiaojin Zhang, Yanhua Wu

**Affiliations:** 1 School of Life Sciences, Human Phenome Institute, Fudan University, Shanghai, China.; 2 The Second Affiliated Hospital, Institute of Biomedical Innovation, Jiangxi Academy of Medical Sciences, Jiangxi Medical College, Nanchang University, Nanchang, China.; 3 Senior Department of Obstetrics and Gynecology, Chinese PLA General Hospital, Beijing, China.; 4 Obstetrics and Gynecology Hospital, Institute of Reproduction and Development, and; 5 National Demonstration Center for Experimental Biology Education, Fudan University, Shanghai, China.

**Keywords:** Genetics, Reproductive biology, Genetic variation

## Abstract

Premature ovarian insufficiency (POI) is a complex reproductive disorder with a strong genetic component. The known POI causative genes currently account for only a small fraction of cases. In this study, we conducted whole-exome sequencing and identified a rare heterozygous missense variant in DNA helicase B (*HELB*) (c.349G>T, p.Asp117Tyr) in a Chinese family with POI and early menopause. To investigate the pathogenicity of this variant, a knockin mouse model carrying a heterozygous missense *Helb* variant (*Helb*^+/D112Y^) homologous to the human *HELB* c.349G>T was constructed. The *Helb*-mutated female mice exhibited reduced litter sizes and prolonged interlitter intervals compared with wild-type mice after reaching 10 months of age, leading to a shortened reproductive lifespan. Consistently, aged *Helb*^+/D112Y^ females showed decreased ovarian weight and accelerated follicle depletion. Transcriptomic analysis of the ovaries from *Helb*-mutated mice revealed dysregulated expression of genes associated with impaired ovarian function and ovarian aging. Collectively, these findings in both humans and mice suggest that *HELB* is involved in maintaining ovarian function and regulating reproductive aging, highlighting the importance of *HELB* in female reproductive health.

## Abstract 2

HELB is involved in regulating female reproductive aging and genetic variants in *HELB* contribute to premature ovarian insufficiency and early age of natural menopause.

## Introduction

Premature ovarian insufficiency (POI) is defined as the occurrence of irregular menstrual cycles (amenorrhea or oligomenorrhea) combined with elevated serum follicle-stimulating hormone (FSH > 25 U/L) measured on 2 occasions at least 4 weeks apart, before the age of 40 (1). The incidence of POI in women is approximately 3.7% (2). POI can result from medical interventions such as chemotherapy, radiotherapy, or surgery, as well as from environmental factors or genetic defects (3–6). Previous studies have reported that 14%–31% of POI cases have a family history and have demonstrated significant correlations in menopausal age between mothers and daughters (7–9), emphasizing the strong genetic basis of POI.

In recent years, the genetic landscape of POI has been expanded by whole-exome sequencing (WES), with approximately 95 POI causative genes identified (10). Notably, most of these genes are enriched in pathways related to DNA damage response (DDR), including meiosis (*STAG3*, *SYCE1*, and *HFM1*) (11–13), double-strand breaks (DSBs) (*PRDM9*, *ANKRD31*, and *RAD51*) (14, 15), homologous recombination (HR) (*SPIDR*, *MCM8*/*9*, and *MSH4/5*) (16–20), and the Fanconi anemia pathway (21–23). On the other hand, POI is a late-onset disease with high heterogeneity in etiology. The big challenge in genetic analysis lies in the fact that each POI causative gene contributed to only a small portion of cases. The genetic defects of most patients with POI in clinic remain to be resolved.

Human DNA helicase B (*HELB*) is located on chromosome 12, belonging to the superfamily 1B (SF1B) DNA helicase, which is highly conserved among vertebrates (24). *HELB* contains 13 exons and encodes a 1,087–amino acid protein, comprising 3 functional domains: a large N-terminal region of unknown function, a central helicase domain, and a C-terminal subcellular localization domain (25). In cells, HELB plays essential roles in maintaining cellular growth and viability by ensuring proper cell cycle progression, facilitating recovery from DNA replication stress, and regulating HR. Knockdown of *HELB* in U2OS cells reduced 5-bromo-2′-deoxyuridine incorporation into newly synthesized DNA, leading to G_1_ phase arrest (26). Similarly, depletion of *HELB* in HeLa cells resulted in decreased cell survival and increased chromosomal aberrations under replication stress (27). HELB is also recruited to single-stranded DNA by interacting with replication protein A (RPA) in response to DSBs (28).

Several population studies implicated the contribution of *HELB* in regulating ovarian aging. In 2015, a genome-wide association study (GWAS) involving 69,360 women of European ancestry identified 3 common variants at the *HELB* locus, 1 synonymous and 2 noncoding (rs3741604, rs1183272, and rs7397861), where the reference allele is associated with later age at natural menopause (ANM) and the alternate allele is associated with earlier ANM (29). They also identified another low-frequency variant (rs75770066) linked to delayed menopause (29). In 2022, another missense variant, rs4430553, was reported to be associated with later ANM (30). More recently, Stankovic et al. performed large-scale exome-wide burden analysis and found that rare, high-confidence, protein-truncating variants in *HELB* (minor allele frequency [MAF] < 0.1%) were associated with delayed menopause (31). Although these studies linked *HELB* to ANM, no animal model–based or molecular mechanistic investigations have been performed to investigate the role of HELB in maintaining ovarian function.

Here we conducted WES on a nonsyndromic POI family and identified a rare heterozygous missense variant in *HELB*, inherited from the mother with early menopause. To investigate its effect, we generated a knockin mouse model and found that the heterozygous *Helb*-mutated mice exhibited subfertility, characterized by age-dependent impairments in fertility and ovarian reserve. Furthermore, RNA-sequencing and transcriptomic analysis revealed significant dysregulation of genes associated with ovarian function in *Helb-*mutated mice.

## Results

### Identifying a heterozygous missense variant of HELB in a Chinese family with POI and early menopause.

**Figure 1:**
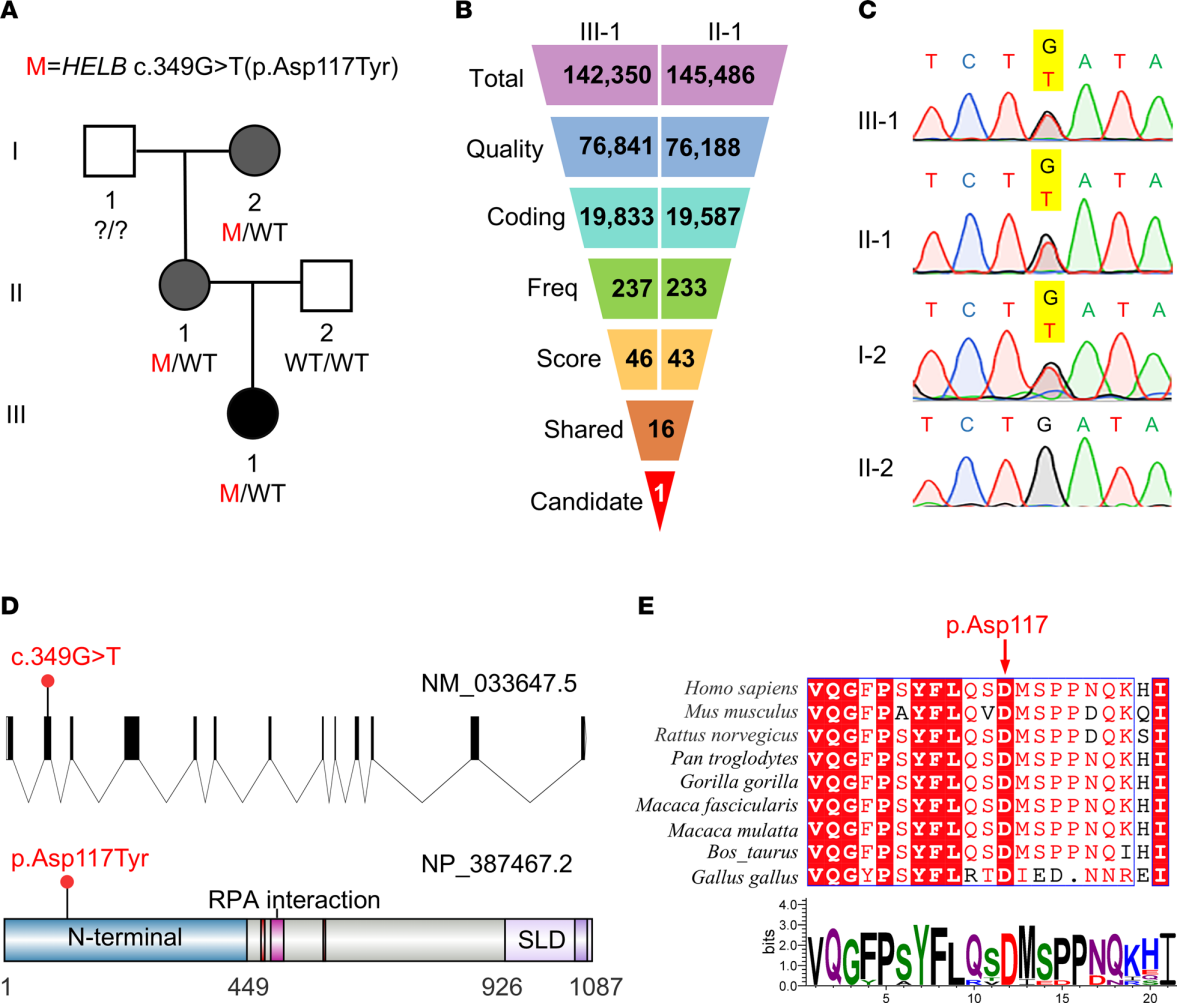
Identification of a deleterious variant of *HELB* in a Chinese family with POI and early menopause. (**A**) A heterozygous missense variant (M) c.349G>T (p.Asp117Tyr) of *HELB* was identified in a Chinese family with POI and early menopause. The black-filled symbol indicates the proband with POI, and gray-filled symbols represent family members affected with early menopause. (**B**) Flowchart of WES filtering strategies based on quality, frequency, deleteriousness, and biological significance. Filters include quality (variants with high/medium calling quality: sequencing depth > 30, genotype quality > 30, no low-quality calls); coding (exonic or splice site variants); Freq (minor allele frequencies < 0.001 in the 1000 Genomes Project [1000g], gnomAD, and ExAC databases); score (variants predicted to be damaging by SIFT, PolyPhen-2, MutationTaster, and CADD_phred > 25); and shared (variants shared by III-1 and II-1). (**C**) Sanger sequencing confirmation of the *HELB* variants in affected individuals. The mutated positions are highlighted in yellow. (**D**) Schematic representation of human genomic *HELB* (NM_033647.5) and human HELB protein (NP_387467.2). The mutated positions are indicated by red dots. (**E**) Conservation analysis of HELB amino acid residues across species. The red arrow indicates the Asp117 residue affected by the identified variant.

The proband in the affected Chinese family (participant III-1, Figure 1A) experienced normal menarche at 14 years of age. After experiencing irregular menstruation for about 1 year, she was diagnosed with POI at the age of 35 years. Her FSH levels were 77.54 IU/L, and an ultrasound examination revealed small ovaries with undetectable follicles (Table 1). The patient had no history of ovarian surgery, chemotherapy, radiotherapy, or autoimmune disease. Premutation or full mutation of the *FMR1* gene was excluded. A family history revealed that the proband’s grandmother and mother (participants I-2 and II-1) both experienced secondary amenorrhea at ages 30 and 38, respectively.

To identify genetic variants associated with POI or early menopause in this family, WES was performed on participants II-1 and III-1. Variants shared between the proband and her mother were considered in the genetic analysis (Figure 1B). Briefly, after excluding low-quality variants and noncoding sequences, we included variants with a MAF < 0.1% in the 1000g, gnomAD, and ExAC databases. Subsequently, nonsense variants, frameshift and non-frameshift indels, splicing variants affecting 1–3 residues at splicing sites, and missense variants predicted to be deleterious by in silico tools (SIFT, PolyPhen-2, MutationTaster, CADD) were retained. This process identified 16 variants in 16 different genes shared by the proband and the mother (Supplemental Table 1; supplemental material available online with this article; https://doi.org/10.1172/jci.insight.191122DS1), but no variant in known POI-causative genes was found. We further reviewed the biological function and expression patterns of all 16 genes and found 1 of the genes, *HELB*, has been reported to be significantly associated with ANM (29–31). Meanwhile, as none of the other 15 genes were found to be linked to ovarian function or ovarian aging in the existing literature (Supplemental Table 1), they were excluded from the analysis. Therefore, a rare missense variant in *HELB* (NM_033647.5, c.349G>T, p.Asp117Tyr) was finally identified, and Sanger sequencing confirmed the presence of this variant in the proband, her mother, and her affected grandmother (Figure 1C).

The *HELB* c.349G>T variant is located in exon 2, within the region coding for an incompletely understood N-terminal domain of HELB (Figure 1D). Conservation analysis across multiple species showed that the Asp117 residue is highly conserved in eukaryotes (Figure 1E). The variant is absent from 1000g, ExAC, and gnomAD databases; rare in PGG.Han database; and predicted to be damaging or deleterious by SIFT, PolyPhen-2, MutationTaster, and CADD (Table 2).

### Shortened reproductive lifespan in the Helb^+/D112Y^ female mice.

**Figure 2:**
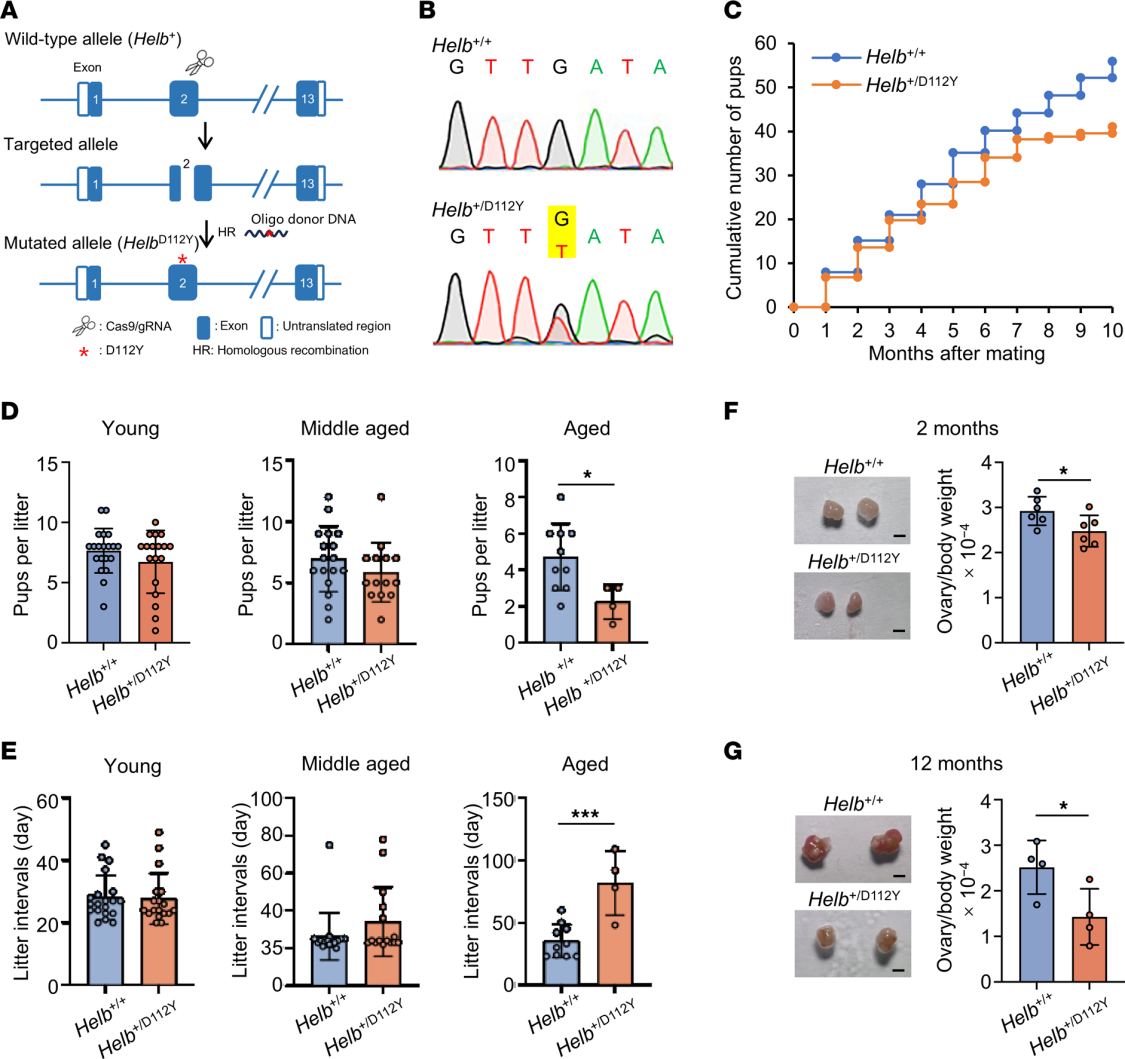
*Helb*^+/D112Y^ knockin mice exhibit reduced female fertility. (**A**) Strategy for generating a knockin mouse model carrying a missense mutation in *Helb* (c.334G>T, p. Asp112Tyr). (**B**) Sanger sequencing confirmation of wild-type (*Helb*^+/+^) and mutant (*Helb*^+/D112Y^) mouse genotypes. The mutated position is highlighted in yellow. (**C**) Cumulative number of pups produced by *Helb*^+/+^ and *Helb*^+/D112Y^ female mice bred continuously with wild-type males for 10 months. *n* = 5. (**D**) Average number of pups per litter for female mice. Mice were categorized into 3 age groups: young (2 to 6 months old), middle-aged (7 to 9 months old), and aged (10 to 12 months old). Data are presented as mean ± SD. *n* = 5. Two-tailed Student’s *t* tests were used for statistical comparisons between 2 groups. *P < 0.05. (**E**) Litter intervals of female mice. Data are presented as mean ± SD. *n* = 5. Two-tailed Student’s *t* tests were used for statistical comparisons between 2 groups. ***P < 0.001. (**F**) Representative images (left) and statistical analysis (right) of ovaries from 2-month-old female mice. Scale bar, 1 mm. Data are presented as mean ± SD. *n* = 6. Two-tailed Student’s *t* tests were used for statistical comparisons between 2 groups. *P < 0.05. (**G**) Representative images (left) and statistical analysis (left) of ovaries from 12-month-old female mice. Scale bar, 1 mm. Data are presented as mean ± SD, *n* = 4. Two-tailed Student’s *t* tests were used for statistical comparisons between 2 groups. *P < 0.05.

To explore the relationship between heterozygous deleterious *HELB* variant and the early onset of ovarian-aging phenotypes observed in our cases, we generated a knockin mouse model using CRISPR/Cas9 technique (Figure 2A and Supplemental Figure 1A). The mutated mouse *Helb*^+/D112Y^ carried a heterozygous missense variant (NM_080446.2, c.334G>T, p.Asp112Tyr), which corresponds to the c.349G>T variant identified in our patients. The introduced variant was confirmed by Sanger sequencing (Figure 2B and Supplemental Figure 1B).

To assess the effect of the *Helb* mutant on female fertility, we first conducted a breeding assay. Adult *Helb*^+/D112Y^ and wild-type female mice (6–8 weeks old) were paired with wild-type C57BL/6 males (8–10 weeks old). The number of pups and the birth dates were recorded continuously over a 10-month mating period. During the first 4 months, the cumulative number of offspring produced by young mutant mice was comparable to that of wild-type mice. However, from the fifth to seventh months, the cumulative number of offspring from middle-aged mutant mice began to decline. By the eighth to tenth months, aged wild-type female mice continued to produce offspring, whereas *Helb*^+/D112Y^ mutant female mice had almost completely lost fertility, with their cumulative pup number remaining nearly unchanged (Figure 2C). Consistently, no markedly significant differences in litter size were observed between young and middle-aged *Helb*^+/D112Y^ females and control groups. However, a statistically significant decline in litter size was evident in aged *Helb*^+/D112Y^ females, with wild-type females producing a mean ± SD of 4.7 ± 1.8 pups per litter compared with only 2.25 ± 1.0 pups in *Helb*^+/D112Y^ females (Figure 2D). Furthermore, the average litter interval was also statistically significantly prolonged in aged *Helb*^+/D112Y^ females, increasing to 81.8 ± 25.8 days compared with 35.3 ± 13.3 days in wild-type females. No significant differences were observed in the young and middle-aged groups (Figure 2E).

Uteruses and ovaries were collected from female mice and subjected to morphological analysis. No anatomical difference was shown in the uteruses between *Helb*^+/D112Y^ and wild-type mice (Supplemental Figure 2). The ovaries of young *Helb*^+/D112Y^ female mice (2 months old) were slightly smaller than those of wild-type female mice (Figure 2F). Consistent with the breeding assay results, the relative ovarian weight of aged *Helb*^+/D112Y^ female mice (12 months old) statistically significantly decreased, with approximately half that of wild-type mice (Figure 2G). Taken together, these findings indicate that the *Helb*^+/D112Y^ heterozygous mutation leads to age-dependent reductions in female fertility and ovarian weight, ultimately resulting in a shortened reproductive lifespan in *Helb*^+/D112Y^ female mice.

### Impaired ovarian function in the Helb^+/D112Y^ female mice.

**Figure 3:**
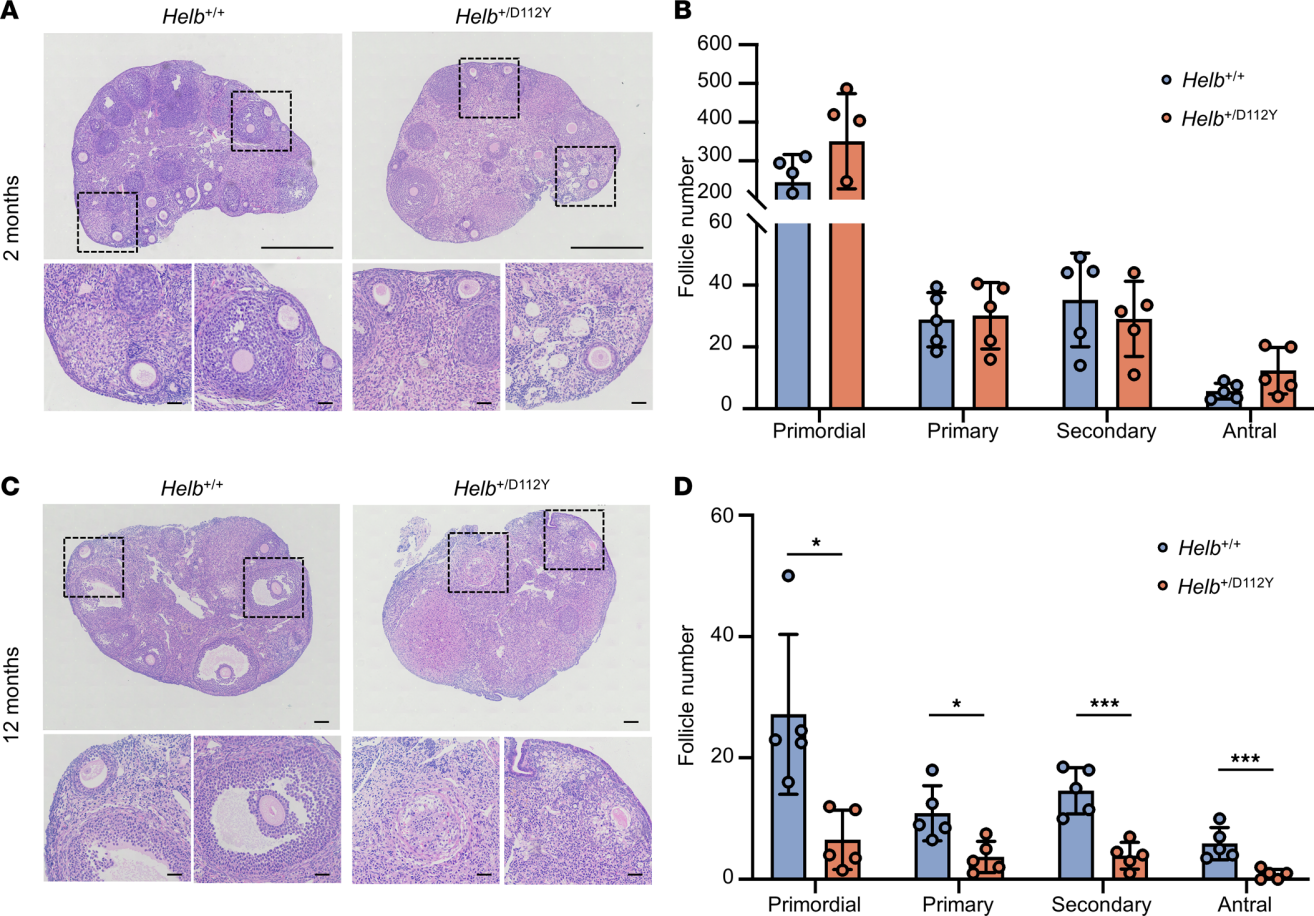
Impaired ovarian function in *Helb*^+/D112Y^ mutant mice. (**A**) H&E staining of ovary sections from 2-month-old *Helb*^+/+^ and *Helb*^+/D112Y^ mice. Scale bar, 100 μm. (**B**) Statistical analysis of the number of primordial, primary, secondary, and antral follicles in 2-month-old *Helb*^+/+^ and *Helb*^+/D112Y^ mouse ovaries. Data are presented as mean ± SD. *n* = 5. (**C**) H&E staining of ovary sections from 12-month-old *Helb*^+/+^ and *Helb*^+/D112Y^ mice. Scale bar, 100 μm. (**D**) Statistical analysis of the number of primordial, primary, secondary, and antral follicles in 12-month-old *Helb*^+/+^ and *Helb*^+/D112Y^ mouse ovaries. Data are presented as mean ± SD. *n* = 5. Two-tailed Student’s *t* tests were used for statistical comparisons between 2 groups. *P < 0.05; ***P < 0.001.

The impact of the *Helb* mutation on female fertility was further evaluated through hematoxylin and eosin (H&E) staining and quantitative analysis of follicle numbers at various stages in ovaries from 2-month-old and 12-month-old mice. The results showed that follicles at all developmental stages, from primordial to antral follicles, were present in 2-month-old *Helb*^+/D112Y^ ovaries (Figure 3, A and B). However, the follicle numbers at all stages were statistically significantly reduced in 12-month-old *Helb*^+/D112Y^ ovaries compared with wild-type ovaries (Figure 3, C and D). With aging, the total number of follicles in 12-month-old wild-type ovaries declined but still retained an average of over 20 primordial follicles and approximately 10 primary and secondary follicles per ovary. In contrast, *Helb*^+/D112Y^ ovaries exhibited only one-fifth the number of primordial follicles and one-third the numbers of primary and secondary follicles observed in wild-type ovaries. In some mutant individuals, follicles at all developmental stages were nearly absent. Consistently, while large antral follicles were observed in wild-type ovaries, they were almost entirely absent in *Helb*^+/D112Y^ ovaries.

**Figure 4:**
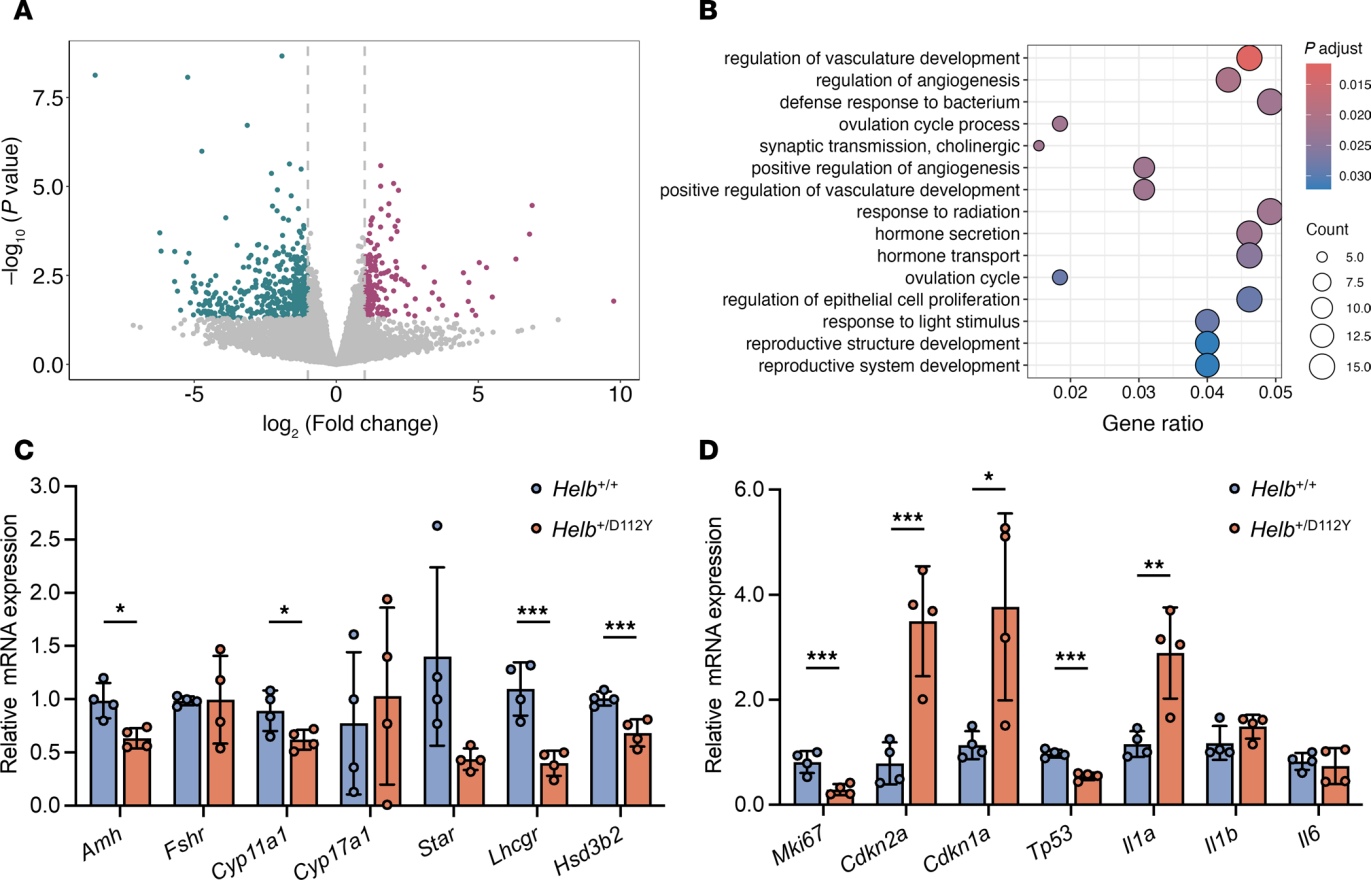
Transcriptomic alterations in *Helb*^+/D112Y^ ovaries. (**A**) Volcano plot showing differentially expressed genes in ovaries from *Helb*^+/D112Y^ mice compared with *Helb*^+/+^ mice at 2 months old. Genes with *P* < 0.05 were marked as red [log_2_ (fold-change) > 1)] or green [log_2_ (fold-change) < 1]. (**B**) GO analysis of downregulated genes in *Helb*^+/D112Y^ mice compared with *Helb*^+/+^ mice. (**C**) The relative mRNA expression levels of *Amh*, *Fshr*, *Cyp11a1*, *Cyp17a1*, *Star*, and *Hsd3b2* in ovaries from *Helb*^+/+^ and *Helb*^+/D112Y^ mice at 2 months old measured by RT-qPCR and normalized to *Gapdh* expression. Data are represented as mean ± SD. *n* = 4. Two-tailed Student’s *t* tests were used for statistical comparisons between 2 groups. *P < 0.05; ***P < 0.001. (**D**) The relative mRNA expression levels of *Mki67*, *Cdkn2a*, *Cdkn1a*, *Tp53*, *Il1a*, *Il1b*, and *Il6* in the ovaries from *Helb*^+/+^ and *Helb*^+/D112Y^ mice at 12 months old measured by RT-qPCR and normalized to *Gapdh* expression. Data are represented as mean ± SD. *n* = 4. Two-tailed Student’s *t* tests were used for statistical comparisons between 2 groups. *P < 0.05; **P < 0.01; ***P < 0.001.

To explore the effect of the *Helb* variant on the transcriptome, ovaries from 2-month-old wild-type and *Helb*^+/D112Y^ mice were collected for RNA sequencing. A total of 188 upregulated genes and 419 downregulated genes were identified in *Helb*^+/D112Y^ ovaries compared with wild-type ovaries (Figure 4A). The enriched biological processes of downregulated genes according to Gene Ontology (GO) enrichment analysis included ovulation cycle, hormone secretion, and reproductive system development (Figure 4B). To validate these findings, real-time quantitative PCR (RT-qPCR) was performed to examine the expression of specific genes involved in these pathways. The expression levels of gonadotropin receptors (*Amh* and *Lhcgr*) and key ovarian steroidogenic enzymes involved in estrogen synthesis (*Cyp11a1* and *Hsd3b2*) were statistically significantly dysregulated in 2-month-old *Helb*^+/D112Y^ ovaries compared with wild-type ovaries (Figure 4C). Since *Helb* has been reported to play roles in cell cycle progression and DDR, we also performed RT-qPCR to analyze gene expression related to these pathways. The results revealed statistically significant downregulation of several key genes related to mitotic cell cycle (e.g., *Blm*, *Cdc14b*, and *Cdkn1b*) and DNA DSB repair (e.g., *Fancm*) in 2-month-old *Helb*^+/D112Y^ ovaries compared with wild-type controls (Supplemental Figure 3). Additionally, aged *Helb*^+/D112Y^ ovaries showed downregulated expression of cell proliferation marker (*Mki67*) and upregulated expression of aging markers (*Cdkn2a*, *Cdkn1a*, *Tp53*, and *IL1a*) compared with wild-type ovaries (Figure 4D). Together, these results indicate that *Helb*^+/D112Y^ mice exhibit impaired ovarian function and early onset of the ovarian aging process, resembling the phenotype of human POI.

## Discussion

In this study, we reported a rare missense variant, *HELB* c. 349G>T, in a Chinese family with POI and early menopause across 3 generations. The human genetic data provided an initial clue and prompted us to explore biological relevance using an in vivo model. We generated knockin mice, *Helb*^+/D112Y^, carrying the homologous mutation, and found that this single nucleotide substitution is sufficient to induce age-dependent subfertility, recapitulating key features of POI in humans.

It is interesting to notice that the *Helb* missense mutation did not impair early reproductive function in young mice but markedly induced early onset of ovarian aging. *Helb*^+/D112Y^ females displayed normal ovarian morphology and fertility in young adulthood but exhibited reduced follicle counts, prolonged litter intervals, and smaller litter sizes starting at 10 months of age. Histological analysis of ovaries from aged mutant mice further revealed marked depletion of ovarian follicles across all developmental stages. These findings closely recapitulate the clinical trajectory of POI observed in our family cohort, where affected women retained reproductive capacity in early adulthood but developed premature ovarian dysfunction by their 30s. At the molecular level, transcriptomic analysis of 2-month-old *Helb*^+/D112Y^ ovaries uncovered marked dysregulation of genes involved in steroidogenesis, gonadotropin response, and aging-related pathways, indicating that ovarian dysfunction initiates at the molecular level prior to overt reproductive phenotypes. These findings in the mice underscore the pivotal role of *HELB* in preserving ovarian function during reproductive aging.

The size of the oocyte pool at birth and the rate of oocyte depletion after sexual maturity determine the age at menopause (32). Genetic studies on human ovarian aging have highlighted the critical role of factors affecting genomic stability in maintaining ovarian function (29, 33, 34). HELB belongs to the SF1B helicase family and was found to participate in DDR by interacting with replication protein A to resolve replication stress and regulate HR (27, 28). By limiting long-range DNA end resection, HELB ensures a balance between HR and nonhomologous end joining repair pathways (28, 35), and disruption of this balance may contribute to ovarian aging. Our identified c.349G>T variant is located in the second exon of *HELB* and substitutes Asp117 with Tyr in the N-terminal region, a domain believed to mediate protein-protein interactions critical for DNA replication and repair (26); whether this variant affects the function of HELB in maintaining genomic stability remains to be explored in the future.

Despite these insights, several limitations in the present studies should be acknowledged. First, although our knockin mouse model provides compelling functional evidence, we did not investigate other human *HELB* variants, especially those missense and nonsense variants associated with delayed menopause. The contrasting effects of different *HELB* variants on ovarian aging remain to be explored in future studies. Second, only a single informative pedigree was used in the WES analysis. Larger cohorts and independent replication studies are needed to establish *HELB* as a causative gene for POI.

Additionally, *HELB* has been implicated in human ovarian aging by previous GWAS conducted in the UK Biobank (29, 30). This convergence prompted us to perform additional analyses in the UK Biobank dataset to further evaluate the role of HELB in reproductive aging (Supplemental Figure 4). Consistent with previous findings, 4 genome-wide significant *HELB* variants, including the previously reported rs75770066 and 3 variants at closely adjacent loci, were associated with delayed menopause (Supplemental Figure 5). Interestingly, we also observed multiple variants that reached FDR-corrected significance (Benjamini-Hochberg–adjusted *P* < 0.05), which exhibited small but opposite effects on ANM (Supplemental Table 2). These variants generally showed low linkage disequilibrium (LD; *r*² < 0.2) with the genome-wide significant cluster, which itself consisted of strongly correlated positive-effect SNPs. Furthermore, colocalization analysis suggested an apparent inverse relationship between *HELB* expression and ANM (Supplemental Table 3); however, significant Heterogeneity in Dependent Instruments tests argued against true colocalization, indicating that these opposing effects likely reflect heterogeneity driven by LD. Together, these population studies support the presence of an LD-driven cluster of positive-effect variants in *HELB*, while the weak negative-effect variants may represent descriptive patterns arising from the complex LD structure rather than independent causal mechanisms. It will nevertheless be important in future studies to further investigate whether different *HELB* variants exert distinct regulatory effects on ovarian function via gene expression.

## Methods

Further information can be found in Supplemental Methods.

### Sex as a biological variable.

This study focused exclusively on female mice because of the investigation of POI and reproductive aging, which are inherently female-specific conditions. The findings are not expected to be directly applicable to males, as *HELB*’s role in ovarian function and menopause timing does not have a clear parallel in male reproductive biology.

### Patients.

The patients initially diagnosed with nonsyndromic POI were recruited at the Obstetrics and Gynecology Hospital of Fudan University. The diagnostic criteria for POI encompassed oligo/amenorrhea for a minimum of 4 months and elevated FSH levels (>25 IU/L) on 2 separate occasions, with at least 4 weeks between assessments, occurring before the age of 40, as recommended (1).

### WES and variant calling.

WES was performed on genomic DNA extracted from the peripheral blood samples of all patients with POI. Variant calling was carried out as previously described (22). Briefly, genomic DNA was captured with the AIExome Human Exome Panel V3 (iGeneTech) and sequenced on the HiSeq X Ten platform (Illumina) with a 150 bp paired-end read length, and then raw data (approximately 10 Gb/exome) were mapped to the human reference genome GRCh37/hg19 using the Burrows-Wheeler Alignment tool (36). Variant calling was performed using the Genome Analysis Toolkit (37), and all variants were further annotated using ANNOVAR software (38). Variants in POI causative/candidate gene sets were subjected to further analysis. Primers for Sanger sequencing of the variant’s identification are listed in Supplemental Table 4.

### Mouse model.

The mouse model was established on C57BL/6J genetic background using CRISPR/Cas9 genome-editing technology by Shanghai Model Organisms Center, Inc. (China). To generate *Helb* mice, guide RNA 5′-CTTTCCGGCATACTTTCTACAGG-3′ was designed to target exon 2 of mouse *Helb* gene (NM_080446.2). All the animals were housed in an environment with a temperature of 22°C ± 2°C, a relative humidity of 60%–65%, and a 12-hour light/12-hour dark cycle and were given basal diet and pure water. Primers for mouse genotyping are listed in Supplemental Table 4.

### Female fertility test.

Sexually mature females of *Helb* mice and controls (6–8 weeks) were used for the fertility test. Female mice were mated with wild-type C57BL/6 males (8–10 weeks) for 10 months. Females were checked for pregnancy daily. The dates of giving birth and the numbers of pups and litters in each cage were recorded for each group.

### RNA extraction and RT-qPCR.

Total RNA was extracted from mouse ovaries with Allprep DNA/RNA/Protein Mini Kit (80004, QIAGEN) and reverse-transcribed into cDNA using Hi-Script RT SuperMix (R423-01, Vazyme), following the manufacturer’s instructions. Quantitative PCR was performed using TB Green Premix Ex Taq (RR820A, TaKaRa) on a CFX Connect Real-Time PCR Detection System (Bio-Rad). The relative target mRNA levels were normalized to expression levels of *Gapdh* and were quantified using the 2^–ΔΔCt^ method. The primers for RT-qPCR analyses are listed in Supplemental Table 5.

### Histological analysis of ovaries and follicle counting.

Ovaries were fixed in 4% paraformaldehyde, dehydrated in graded alcohol and xylene, and embedded in paraffin. Paraffin-embedded ovaries were serially sectioned at 5 μm H&E staining. The follicles in ovarian sections were classified into primordial, primary, secondary, and antral follicles according to their morphology based on standards Pedersen established (39). Briefly, primordial follicles have an oocyte surrounded by a single layer of squamous granulosa cells; primary or secondary follicles have an oocyte surrounded by a single layer or several layers of cuboidal granulosa cells, respectively; and antral follicles have a large oocyte with many layers of granulosa cells and with an antrum. Only the follicles that contained oocytes with clearly visible nuclei were counted. Every fifth section was selected for evaluation and then multiplied by 5 to compute the total number of follicles.

### RNA sequencing.

A total of 1–2 μg of ovarian RNA per mouse was used for library preparation. The mRNA libraries were generated using the TruSeq RNA Kits (Illumina) following the manufacturer’s protocol. Index codes were added for sample identification. Libraries were sequenced on the HiSeq X Ten.

### Data quality control and analysis.

FastQC and FASTX_Toolkit were used for quality control, removing sequences with quality scores less than 20 and trimming adapter sequences for small RNA data. Kallisto (v0.44.0) quantified mRNA expression. Differential expression analysis was performed with DESeq2, using FDR-adjusted *P* values less than 0.05 as thresholds for identifying differentially expressed genes. Pathway analysis of differentially expressed mRNAs was done using clusterProfiler and GO databases.

### Statistics.

Comparisons of quantitative data were performed by 2-tailed Student’s *t* test. *P* < 0.05 was considered significantly different, and *P* < 0.01 was considered extremely significantly different.

### Study approval.

This study was conducted in accordance with the Declaration of Helsinki and approved by the institutional review boards of Fudan University Obstetrics and Gynecology Hospital (2017–2019).

### Data availability.

The raw sequence data reported in this work are available under accession number CRA029842, which is publicly accessible at Genome Sequence Archive: https://bigd.big.ac.cn/gsa All data values of the figures are provided in the Supporting Data Values file. Additional information is available upon request from the corresponding author.

## Author contributions

YP conceptualized the study, performed the experiments, conducted data analysis, and wrote and edited the original draft. Y Yu and JM performed experiments, conducted data analysis, and edited the manuscript. SR and ZZ conducted data analysis. XY and YL performed the experiments. FZ acquired funding, conceptualized the study, administered and supervised the project, and reviewed the writing. Y You conceptualized the study and administered and supervised the project. XZ provided resources and administered and supervised the project. YW acquired funding, conceptualized the study, administered and supervised the project, and reviewed and edited the writing.

## Funding support

- National Key Research and Development Program of China (2022YFC2703800 to YW).
- National Natural Science Foundation of China (32270658 to YW and 32288101 to FZ).

## Supplementary Material

Supplemental data

Supplemental tables 1-5

Supporting data values

## Figures and Tables

**Table 1:**
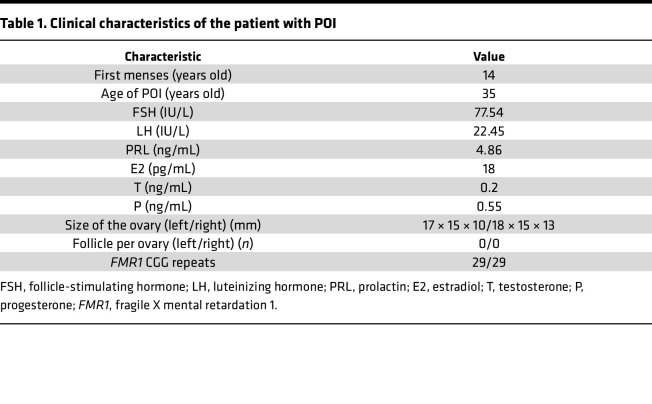
Clinical characteristics of the patient with POI

**Table 2:**
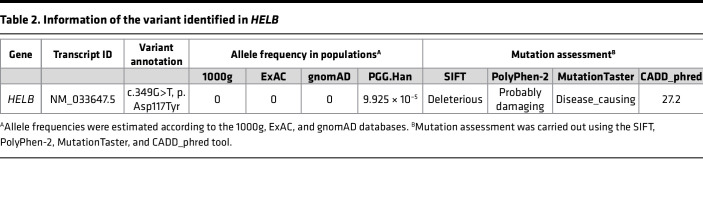
Information of the variant identified in *HELB*

## Version 1 11/10/2025

Electronic publication
