# Supplementary material for: Genetic variants in HELB contribute to premature ovarian insufficiency and early age of natural menopause
Source: JCI Insight. 2025 Nov 10;10(21):e191122. doi: 10.1172/jci.insight.191122 (PMC12643492; doi:10.1172/jci.insight.191122)
Supplement: Supplemental data [file jciinsight-10-191122-s050.pdf]

Genetic variants in *HELB* contribute to premature ovarian insufficiency and early age of natural menopause

Yuncheng Pan, *et al*

Supplementary Information

The supplementary information consists of 5 supplementary figures and supplementary methods. Five supplementary tables are attached separately.

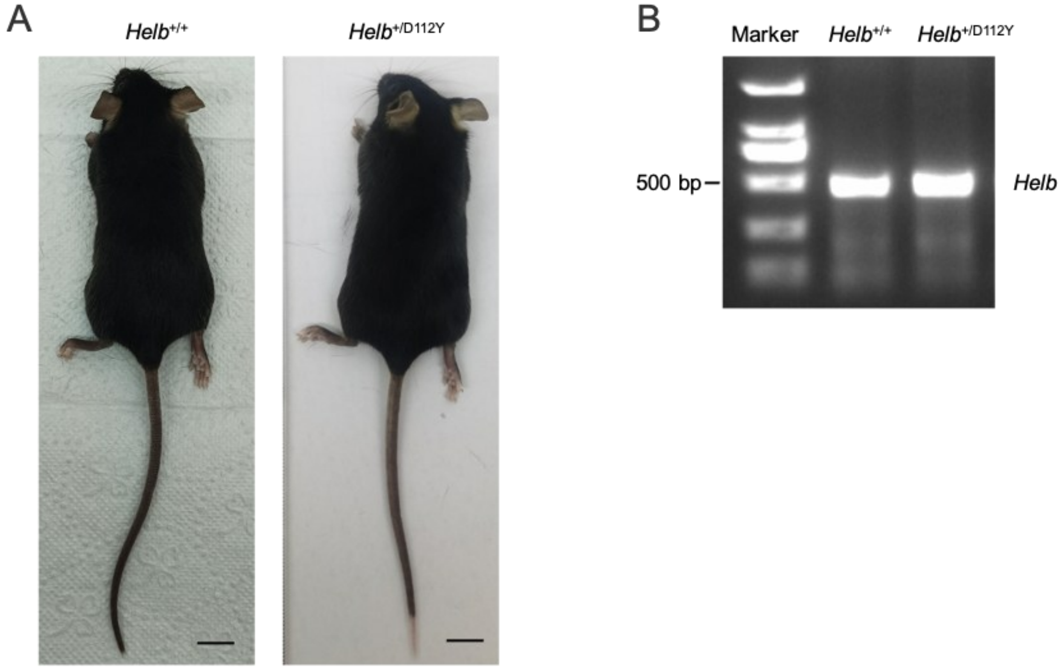

Supplemental Figure 1. Generation of the knock-in mouse model. (A) Representative images of 2-month-old *Helb*$^{+/+}$ and *Helb*$^{+/D112Y}$ female mice. Scale bar, 1 cm. **(B)** PCR genotyping result of *Helb*$^{+/+}$ and *Helb*$^{+/D112Y}$ mice. DNA were obtained from tail samples of 4-week-old mice.

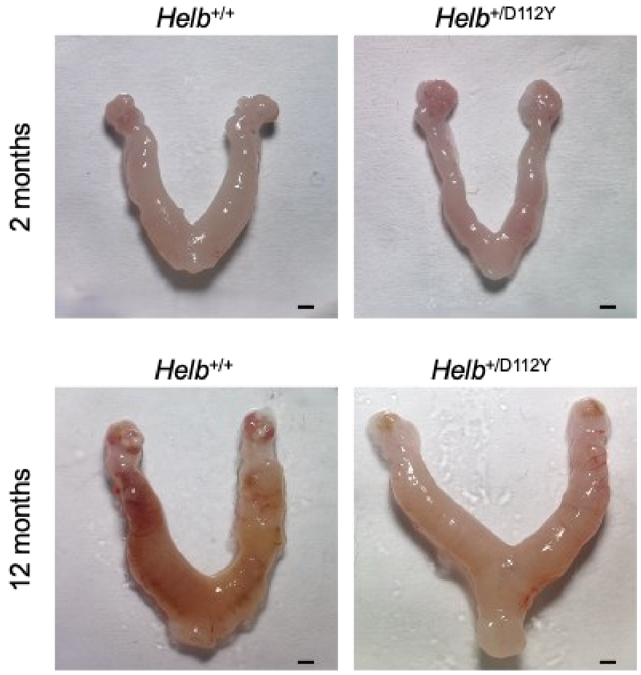

Supplemental Figure 2. Representative images of uteruses from 2-month-old and 12-month-old female mice of. Scale bar, 1 mm.

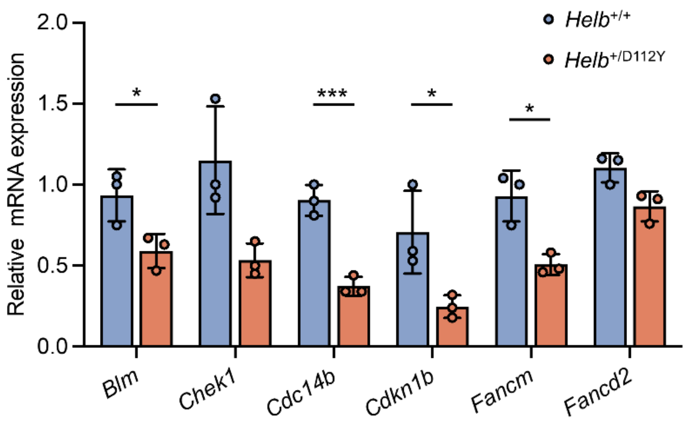

Supplemental Figure 3. The relative mRNA expression levels of genes related to cell cycle and DNA damage repair in *Helb*$^{+/D112Y}$ ovaries. The relative mRNA expression levels of *Blm*, *Chek1*, *Cdc14b*, *Cdkn1b*, *Fancm* and *Fancd2* in ovaries from *Helb*$^{+/+}$ and *Helb*$^{+/\mathrm{D112Y}}$ mice at 2 months old measured by RT-qPCR and normalized to *Gapdh* expression. Data are represented as mean ± SD, $n = 3$. Two-tailed Student's t tests were used for statistical comparisons between 2 groups. *, $P < 0.05$; ***, $P < 0.001$.

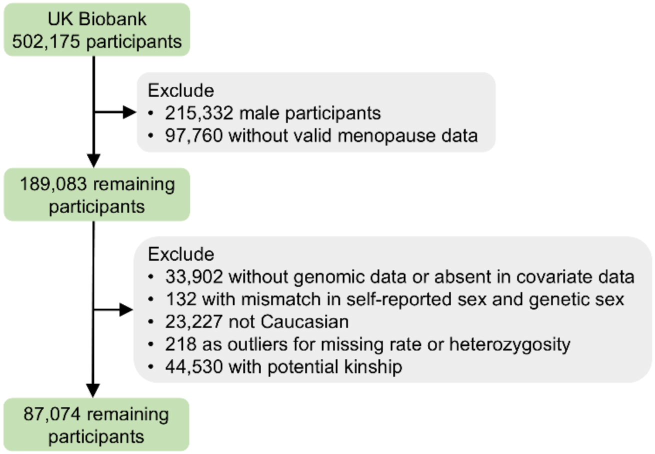

Figure S4. Flowchart of the GWAS analysis on age at menopause in European ancestry female from the UK Biobank dataset.

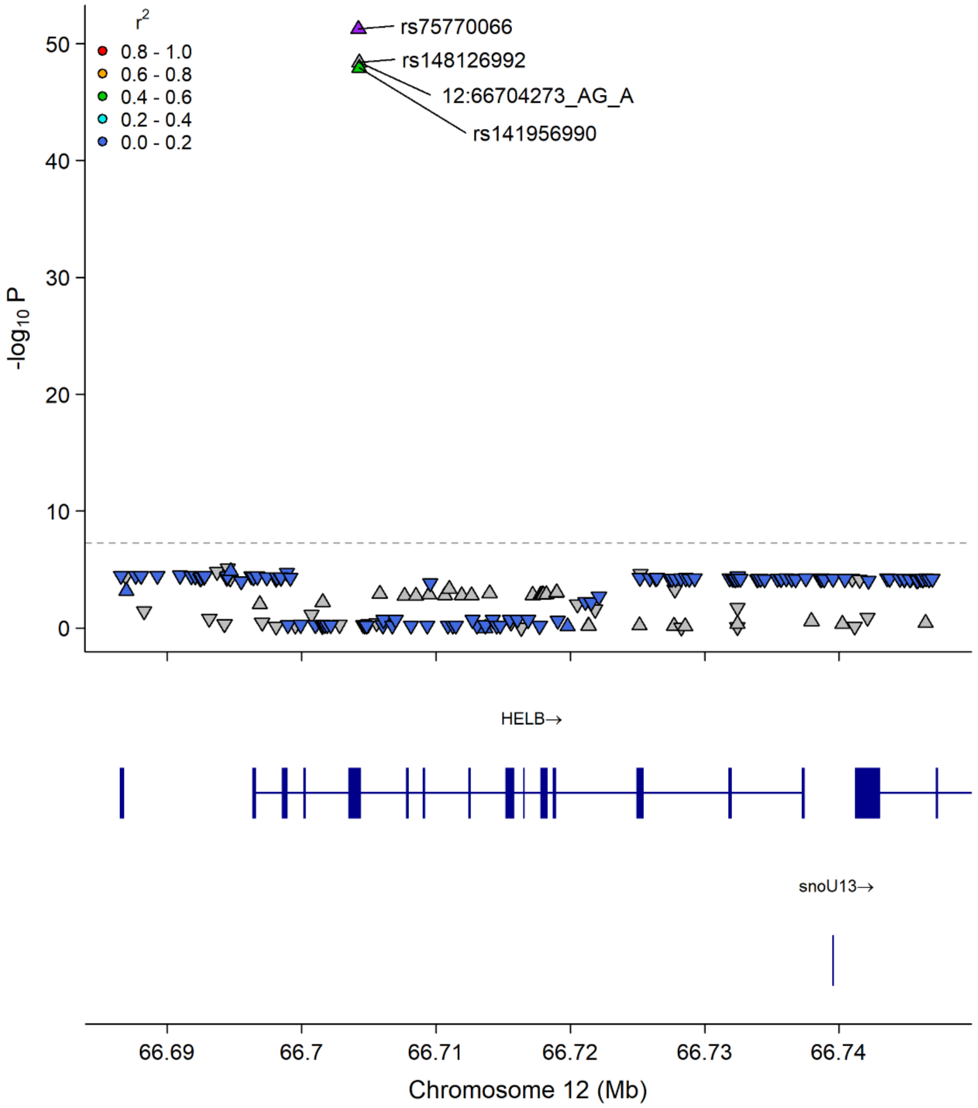

Figure S5. Regional association plot of the *HELB* locus for age at menopause in the UK Biobank. Dot colors were based on 1000 Genome LD score. The rsID of genome-wide significant variants were labelled.

Supplementary Methods

Genome-wide association study. Genetic data were obtained by the UK Biobank, a large prospective cohort study of over 500,000 participants aged 40-69 years, recruited between 2006 and 2010 across 22 assessment centers in the UK (UKB application Number 103791). Genotyping was performed using the UK BiLEVE array and the UK Biobank Axiom array. Detailed protocols for array design, quality control (QC), and imputation have been described elsewhere (1). Moreover, we removed individuals who did not meet the study criteria, and finally remained 87,074 unrelated female participants of European ancestry for analysis. All analyses were conducted in September 2024.

Variant-level QC was performed separately for array and imputed variants within the *HELB* gene region ± 10kb (Chr12: 66,686,325 - 66,747,423 bp, GRCh37). For array-based variants, those with a MAF < 0.01, call rate < 0.95, or Hardy-Weinberg equilibrium (HWE) P value > 1×10^{-6} were excluded. For imputed variants, those with an imputation INFO score < 0.7 or MAF < 0.01 were exlcuded. All QC procedures were assessed using PLINK 2.0 and qctool v2.

Genetic associations with ANM were assessed using the Bayesian mixed-model association test implemented in BOLT-LMM v2.4.1. Regression models were adjusted for age at recruitment and the first 10 principal components to account for population stratification. The Benjamini-Hochberg (BH) procedure was applied to control the false discovery rate (FDR). Variants with P < 5×10^{-8} were considered genome-wide

significant, while those with BH-adjusted P values (PBH) < 0.05 were considered FDR-significant.

Post-GWAS. To elucidate variant-to-gene relationships and potential functional relevance, FDR-significant variants were annotated using ANNOVAR. Linkage disequilibrium (LD) information was obtained from the LDlink API, based on the European population of the 1000g Project. Regional association plot was generated using the locuszoomr R package (v0.3.8), incorporating LD structure and gene annotation from Ensembl v75 (GRCh37). Additionally, colocalization with multi-dimensional QTLs was applied using the Summary-data-based Mendelian Randomization (SMR) portal (2, 3).

Reference

1. Bycroft C, et al. The UK Biobank resource with deep phenotyping and genomic data. *Nature.* 2018;562(7726):203-9.
2. Zhu Z, et al. Integration of summary data from GWAS and eQTL studies predicts complex trait gene targets. *Nat Genet.* 2016;48(5):481-7.
3. Guo Y, et al. SMR-Portal: an online platform for integrative analysis of GWAS and xQTL data to identify complex trait genes. *Nat Methods.* 2025;22(2):220-2.
